# Hematopoietic Stem Cell Factors: Their Functional Role in Self-Renewal and Clinical Aspects

**DOI:** 10.3389/fcell.2022.664261

**Published:** 2022-03-24

**Authors:** Zoya Mann, Manisha Sengar, Yogesh Kumar Verma, Raja Rajalingam, Pawan Kumar Raghav

**Affiliations:** ^1^ Independent Researcher, New Delhi, India; ^2^ Department of Zoology, Deshbandhu College, University of Delhi, Delhi, India; ^3^ Stem Cell and Gene Therapy Research Group, Institute of Nuclear Medicine and Allied Sciences (INMAS), Delhi, India; ^4^ Immunogenetics and Transplantation Laboratory, Department of Surgery, University of California San Francisco, San Francisco, CA, United States

**Keywords:** Hematopoietic Stem Cells (HSCs), hematopoiesis, self-renewal, differentiation, transcription factors, extracellular vesicles (EVs), Graft-versus-Host Disease (GvHD), miRNAs

## Abstract

Hematopoietic stem cells (HSCs) possess two important properties such as self-renewal and differentiation. These properties of HSCs are maintained through hematopoiesis. This process gives rise to two subpopulations, long-term and short-term HSCs, which have become a popular convention for treating various hematological disorders. The clinical application of HSCs is bone marrow transplant in patients with aplastic anemia, congenital neutropenia, sickle cell anemia, thalassemia, or replacement of damaged bone marrow in case of chemotherapy. The self-renewal attribute of HSCs ensures long-term hematopoiesis post-transplantation. However, HSCs need to be infused in large numbers to reach their target site and meet the demands since they lose their self-renewal capacity after a few passages. Therefore, a more in-depth understanding of *ex vivo* HSCs expansion needs to be developed to delineate ways to enhance the self-renewability of isolated HSCs. The multifaceted self-renewal process is regulated by factors, including transcription factors, miRNAs, and the bone marrow niche. A developed classical hierarchical model that outlines the hematopoiesis in a lineage-specific manner through *in vivo* fate mapping, barcoding, and determination of self-renewal regulatory factors are still to be explored in more detail. Thus, an in-depth study of the self-renewal property of HSCs is essentially required to be utilized for *ex vivo* expansion. This review primarily focuses on the Hematopoietic stem cell self-renewal pathway and evaluates the regulatory molecular factors involved in considering a targeted clinical approach in numerous malignancies and outlining gaps in the current knowledge.

## 1 Introduction

The bone marrow resident hematopoietic stem cells (HSCs) direct the process of hematopoiesis (Rieger and Schroeder, 2012). The earliest experimental evidence of the HSCs function was reported when the transplantation of bone marrow (BM) into irradiated mice gave rise to myeloid lineage cells within the spleen of the transplanted mice (Bertrand et al., 2010). The resultant restoration of the differentiated blood cell population was a presupposed function of the HSCs by their stemness, self-renewal property, and asymmetric cell division (Post and Clevers, 2019). Studies revealed that the HSCs rich niches, include BM, peripheral blood, umbilical cord, or fetal liver (Cheng et al., 2020).

Fetal BM HSCs were isolated for the first time in 1992, disclosing their property of asymmetric cell division upon functional characterization (Cheng et al., 2020). HSCs divide into two daughter cells with similar properties: long-term HSCs (LT-HSCs) and short-term (ST-HSCs) (Yang et al., 2005). LT-HSCs are a population of quiescent cells residing in the BM with a self-renewal capacity retained for greater than 6 months (Cheng et al., 2020). However, ST-HSCs are lineage-committed, depending on the niche’s intrinsic and extrinsic signals, and thus cannot sustain their self-renewal property for more than a month (Cheng et al., 2020). ST-HSCs differentiate into hematopoietic progenitor cells (HPCs), which further segregate into common myeloid progenitors (CMPs) and common lymphoid progenitors (CLPs). The CMPs give rise to granulocyte-macrophage progenitors (GMPs) and megakaryocyte-erythrocyte progenitors (MEPs). The GMPs form granulocytes, monocytes, and dendritic cells, while MEPs transform into erythrocytes and megakaryocytes. The CLPs, on the other hand, give rise to T, B, NK, and dendritic cells (Cheng et al., 2020).

Genetically modified mouse models are commonly used to delineate the hematopoietic niche and specific HSC populations because of the ability to control the time and location of somatic mutation in such models. Tie2^−^ Cre model is a common *in vivo* fate mapping system specific to HSCs wherein the Tie2 gene was manipulated using the Cre/loxP system. The Tie2^+^ labeled LT-HSCs amplify the ST-HSCs and multipotent progenitors (MPPs) populations validating the spot of LT-HSCs at the top of the lineage map (Höfer and Rodewald, 2018). The progenitor cells lack self-renewal potency, observed in transplantation of donor stem or progenitor cells in myeloablated mice, wherein these transit-amplifying cell types were unable to restore hematopoiesis completely (Höfer and Rodewald, 2018; Busch et al., 2015). However, further quantification revealed that these progenitors possess self-renewal potential lesser than that of LT-HSCs, in addition to their transiting nature (Höfer and Rodewald, 2018; Busch et al., 2015). However, myeloid progenitor cells lack self-renewal since it is tightly regulated by various endogenous molecules, including transcription factors (TFs), cytokines, niche conditions, and epigenetic modifiers (Table 1) (Sun et al., 2014; Busch et al., 2015; Höfer et al., 2016; Raghav and Gangenahalli, 2018). Mx1-Cre is another inducible tool in which Cre recombinase is activated under the Mx1 promoter by either synthetically designed dsRNA that induces production of interferons IFN-α and IFN-β, or other immunostimulants like polyinosinic: polycytidylic acid (pIpC) (Joseph et al., 2013). This immunostimulation is specific to HSCs that have been contrived to visualize the perivascular cells located close to the endosteum, and the involvement of proteins like Ash2l required to differentiate early hematopoietic progenitor cells by epigenetic regulation (Lüscher-Firzlaff et al., 2019). Nonetheless, it does not have as wide-scale application as Tie2^−^ Cre since this system is usually induced by IFN cytokine and has been reported as a low detectable rate of Cre induction by the endogenous IFN levels itself. It is also known to change the immunophenotype of HSCs transiently thus has been disregarded as a standard tool (Velasco-Hernandez et al., 2016). Additionally, Vav-Cre Vav-iCre strains have been exploited to study gene deletions in the hematopoietic system. Vav gene in the mice encodes for the GEF protein, expressed almost exclusively in the adult HSCs. However, the Vav gene also shows some expression levels in the testicular germ cells and placental trophoblasts. Therefore, in addition to the Vav-Cre strain, a codon improved Vav-iCre model was developed, which is less susceptible to epigenetic silencing [11]. Locking the Vav sites with a GFP gene introduced a new Vav-GFP transgenic line which selectively labels only the nucleated HSCs population. The role of Runx1 and Atg-7 driven autophagy in HSCs homeostasis was hence determined (Speck and Iruela-Arispe, 2009; Hashimoto et al., 2021).

**TABLE 1:** Proteins/genes, their impact on self-renewal, and their interaction with other proteins/genes involved in regulating hematopoiesis.

Proteins/genes	Impact on self-renewal	Interacting proteins/genes	PMID
Angptl3 (Angiopoietin-like protein-3)	Increases	Ikaros	20959605
β−catenin	Increases	Notch1, HOXB4	18371452, 26344907
Bmi-1 (B cell-specific moloney murine leukemia virus integration site 1)	Increases	p16^Ink4a^, p19Arf, Ezh1, Ezh2	16954369, 12714971
Bmp4 (Bone morphogenetic protein 4)	Increases	p38 MAPK, MITF, ITGA4	23243277, 19759357
C/EBPa (CCAAT enhancer binding protein-a)	Decreases	N-Myc	23502316
CaR (Calcium-sensing receptor)	Increases	—	16382241
Catalase	Increases	—	25603016
Cdh2 (Cadherin-2)	Increases	Ctnnb1	22901259, 20207221
Cdkn1a or p21 (Cyclin dependent kinase inhibitor 1a)	Increases	Dkk1, NF-Y, Skp2, Ptpmt1, Gfi1, Egr1	10710306, 18397757, 15457180, 23290137, 21502543, 18371452, 22072554
Cdkn1c or p57 (Cyclin dependent kinase inhibitor 1c)	Increases	p27, Ccnd2, Ccnd1, Cdk4, Cdk6	21885020, 21885021
Cdkn2c or p18 (Cyclin dependent kinase inhibitor 2c)	Decreases	p21, p18	15122268
c-Kit	Increases	Bcl-2, SCL and SCF, PI3K/MAPK	18250409, 24446491, 1721869
c-Mpl	Increases	TPO/JAK2	9448308, 18618018, 18371409
c-Myb	Increases	PU.1, b-Myb	24516162, 20823231, 19955420
CREBBP/Cbp (CREB binding protein)	Increases	Ccnd1, Ccnd2, p21, Rb1, Mcm7, Gfi1b, Cxcl12	22006020
Dmtf1 (Cyclin D binding Myb like transcription factor 1)	Decreases	p21, p57, Cdkn2a, Ccnd1, Junb, Cdk6	22039255
Dkk1 (Dickkopf-1)	Decreases	Cdkn1a (p21Cip1)	18371452
Egr1 (Early growth response 1)	Increases	Bmi1, Cdk4, p21	18397757
Erdr1 (Erythroid differentiation regulator 1)	Increases	Fos, Tcfec, Sfpi1, Hmgb	19379700
Erg (ETS-related gene)	Increases	c-Mpl/TPO	21673349
Evi-1 (Ecotropic viral integration site-1)	Increases	GATA-2, Pbx1, Runx1, TGF-β	29658164, 12032771,24573248
Ezh1 (Enhancer Of Zeste 1 Polycomb Repressive Complex 2 Subunit)	Increases	Cdkn2a, Bmi1, Igfbp3, IL-6, p21, Zmat3, Bmp2, p16^Ink4a^	23122289
Ezh2 (Enhancer of zeste 2 polycomb repressive complex 2 subunit)	Increases	HOX isoforms	16293602
FoxO3	Increases	Cdkn1b, p57, Catalase	18371339
GATA-3	Increases and decreases	p38 MAPK, GFI1, MEF/ELF4, FoxO3A, GFI1B, EGR1, JUNB	19377048, 22267605, 23974957
Gfi1 (Growth factor independent 1 transcriptional repressor)	Increases	Mysm1, GATA-2, p21, RUNX1	15457180
Hes1 (Hairy and enhancer of split 1)	Increases	RBP, Notch1	12406868
HIF-1α (Hypoxia inducible factor 1α)	Increases	p16^Ink4a^/p19(Arf)	20804974
HOXB4 (Homeobox B4)	Increases	—	7622039
IGFBP2 (Insulin Like growth factor binding protein 2)	Increases	Cdkn2a, PTEN, p19, p57, p21, p16	21821709
Ikzf1	Decreases	Hes1	20959605
Itch (Itchy E3 ubiquitin protein ligase)	Decreases	Notch1	21478879
Jagged-1	Increases	Vcam, Hes1, Hey1, Notch	24012753
Keap (Kelch Like ECH associated protein 1)	Increases	Nrf2	28674188
Klf10 (Kruppel Like Factor 10)	Increases	Fos, Tcfec, Sfpi1, Hmgb	19379700
Meis1	Increases	HIF-1α, Pbx1	23091297
MK2 or MAPKAPK2 (MAPK Activated Protein kinase 2)	Increases	Edr1/2, Phc1, Phc2	19369945
NF-Ya (Nuclear transcription factor Y-α)	Increases	Notch1, Hes1, p21, Bmi1, Bcl-2, HOX4, LEF-1	22072554, 16081537
Notch1	Increases	Satb1, Jagged-1, Itch	16151515, 21478879, 11895769
Nrf2 (Nuclear factor erythroid 2-related factor 2)	Increases	CXCL12/CXCR4, Ccnd1, PI3K/Akt	23434824
p38 MAPK	Decreases	Ask1, Cdkn2a	16565722
Pbx-1	Increases	P21 and p57, TGF-β, MAPK	24573248
Pcgf2 (Polycomb group ring finger 2)	Decreases	HOXB4	15183898
Pdk2, Pdk4 (Pyruvate dehydrogenase kinase)	Increases	HIF-1α	23290136
Pld3 (phospholipase D family member 3)	Increases	—	19377048
Prnp (Prion Protein)	Increases	—	19377048
PTEN (phosphatase and TENsin homolog deleted on chromosome 10)	Increases	Skp2, mTOR	21931116, 16598206
Ptpmt1 (phosphatidylglycerophosphatase and protein-tyrosine phosphatase 1)	Increases	p21, p57)	23290137
PU.1	Increases	Gfi1, p21, p57, Cdk1, Cdc25a	23395001, 24573248
Rhob (Ras Homolog Family Member B)	Increases	VWF, Pld3	19377048
Sall4 (Spalt like transcription factor 4)	Increases	Epigenetics controlling proteins	22555391, 22150312
SATB1 (Special AT-rich sequence binding protein 1)	Increases	Sfpi1, Nanog, Numb, Myc	23563689
SCF (Stem cell factor)	Increases	Dpt, CXCL12, TPO, IL-3, IL-6	27365423, 11067877, 25100529
SCL (Stem cell leukemia)	Increases	c-Kit	19850742, 14726374
SFRP1 (Secreted Frizzled Related Protein 1)	Increases	Ccnd1 and Dkk1, Pparg, Hes1, Runx1	19664990
SH2B3 (SH2B adaptor protein 3)	Decreases	TPO/Mpl/JAK2/Lnk	17284614, 18618018
Sirt1 (Sirtuin 1)	Increases	Trp53, HOXA9	23630229
Sirt3 (Mitochondrial sirtuin 3)	Increases	SOD2	23375372
Skp2 (S phase kinase-associated protein-2)	Decreases	PTEN, p21, Cdkn1b, Ccnd1, Rbl2, Mad1, p27, p57	21502543, 21931116, 15654333, 21885020
Slug or SNAI2	Decreases	Cyclin D1, p21	20032500
SOD (Superoxide dismutase)	Increases	GSH, Catalase	24605290
STAT5 (Signal transducer and activator of transcription 5)	Increases	Gab2, TPO/c-Mpl, Tie-2, p57	20161778, 19258595
Tal1 (T-cell acute lymphocytic leukemia protein 1.)	Increases	SCL, p21, Id1	19850742
Tdgf1 (Teratocarcinoma-derived growth factor 1)	Increases	Cripto/GRP78	21982233
TNF (Tumor necrosis factor-α)	Decreases	Tnfrsf1a, Tnfrsf1b	21768269
TPO (Thrombopoietin)	Increases	c-Mpl, p57, Ang-1, c-Myc, Tie2	12163458, 18371409, 9892696
Vps72 (Vacuolar protein sorting 72 Homolog)	Increases	Fos, Tcfec, Sfpi1, and Hmgb	19379700
Vwf (Von willebrand factor)	Increases	Rhob, Pld3	19377048
Wilf1 (Wnt inhibitory factor 1)	Decreases	Shh, Jagged-1, Cdh2/N-cadherin, Cxcl12, Bmp4	21652676

Blood cancers account for 10% of all cancer cases. Any disruption in HSCs functioning leads to various hematological malignancies such as leukemia, myeloma, and lymphoma. These three cancer types can be further categorized into acute myeloid leukemia (AML), B-cell acute lymphoblastic leukemia (B-ALL), T-cell ALL (T-ALL), myelodysplastic syndrome (MDS), myeloproliferative neoplasm (MPN), chronic lymphocytic leukemia (CLL), and follicular lymphoma (FL) (Taylor et al., 2017).

Leukemic growth and HSCs maintenance is also regulated by the metabolic pathways. Among the 22 amino acids, leukemic niche is heavily dependent on amino acid metabolism for sustenance of leukemic stem cells (LSCs), unlike HSCs which are only affected negatively by valine deficiency (Tabe et al., 2019). The niche cells secrete certain amino acids that modulate the functioning of the HSCs and LSCs. Understanding of these metabolic patterns is fundamental to pave a way to amino-acid based leukemia targeting therapies. For instance, L-asparaginase shows excellent results in acute lymphoid leukemia (ALL) treatment. The stromal cells secrete asparagine and cysteine which support tumor metabolism as well as glutamine secretion by adipocytes. These mechanisms are considered important in TCA cycle and activation of NK and T cells (Tabe et al., 2019). Thus, their respective inhibitors asparaginase, cysteinase and GLS inhibitor have shown promising clinical outcomes in amino acid depletion and hence, tumor suppression (Fultang et al., 2021). However, a recent study established that only the LSCs are dependent on amino acid metabolism for their functionality and survival (Jones et al., 2018). Similarly, pleiotrophin, a heparin binding growth factor secreted by the human brain and marrow sinusoidal endothelial cells, supports LT-HSCs expansion and plays a critical role in self-renewal (Himburg et al., 2012). Additionally, HSCs also rely on fatty acid oxidation (FAO) for asymmetric cell division since disrupting this pathway causes loss of HSCs quiescence and enhanced differentiation leading to exhaustion of HSCs pool (Miyamoto et al., 2007; Ito and Suda, 2014; Ito et al., 2016). Peroxisome proliferator-activated receptor δ (PPARδ) acts as a transcription factor for FAO regulators and its suppression decreases ATP levels and drives HSCs more towards differentiation. Additionally, Pink/PARKIN mediate autophagy within HSCs to control FAO rates and thus, their maintenance by self-renewal and quiescence.

The self-renewability of HSCs is exploited in this aspect to serve as a therapeutic strategy to restore hematopoietic hierarchical progenitors via myeloid, lymphoid, and erythroid intermediates. LT-HSCs derived from BM, peripheral blood, or umbilical cord are used in transplantation. However, direct aspiration of these tissue-specific HSCs is considered invasive, and the doses administered to the patients need to be standardized case to case (Morrison et al., 1995; Cheng et al., 2020). Therefore, *ex vivo* expansion strategies to enhance the self-renewal and proliferation of HSCs need to be devised. For instance, it has been shown that stem cell factor (SCF) and Src homology 2 domain-containing protein tyrosine phosphatase 1 (SHP-1) inhibitor, 8-Hydroxy-7-(6-sulfonaphthalen-2-yl)diazenyl-quinoline-5-sulfonic acid, Disodium Salt (NSC87877) synergism activates c-Kit and inhibits c-Kit negative regulators (SHP-1/SHP-2) that can be used clinically to enhance cellular proliferation of both erythroid and megakaryoblast cells (Raghav et al., 2018a; Raghav et al., 2018b).

Moreover, extracellular vesicles (EVs) are 30 to 10,000 nm sized cargo encapsulating lipid bilayers, and can be further categorized into exosomes and microvesicles based on their size. HSCs secreted exosomes typically fall within the range 30–120 nm (Grenier-Pleau and Abraham, 2021). The EVs secreted by mesenchymal stem cells (MSCs) are cargoes for miRNAs and piRNAs, which regulate hematopoietic processes by HSCs uptake. IL-4 stimulated macrophages secrete EVs enriched with miRNA-99a/146b/378a, which reduce inflammation by NF-κB and TNF-α targeting and hence, also restrict HPCs expansion. Also, AML-derived EVs suppress HPCs clonogenicity since they are enriched with DKK-1, IL-6 and CCL3 reponsible for leukemic growth and disease progression. Thus, EV-based therapies is another new direction of clinical therapies which is being better understood these days for hematological malignancies.

**FIGURE 1:**
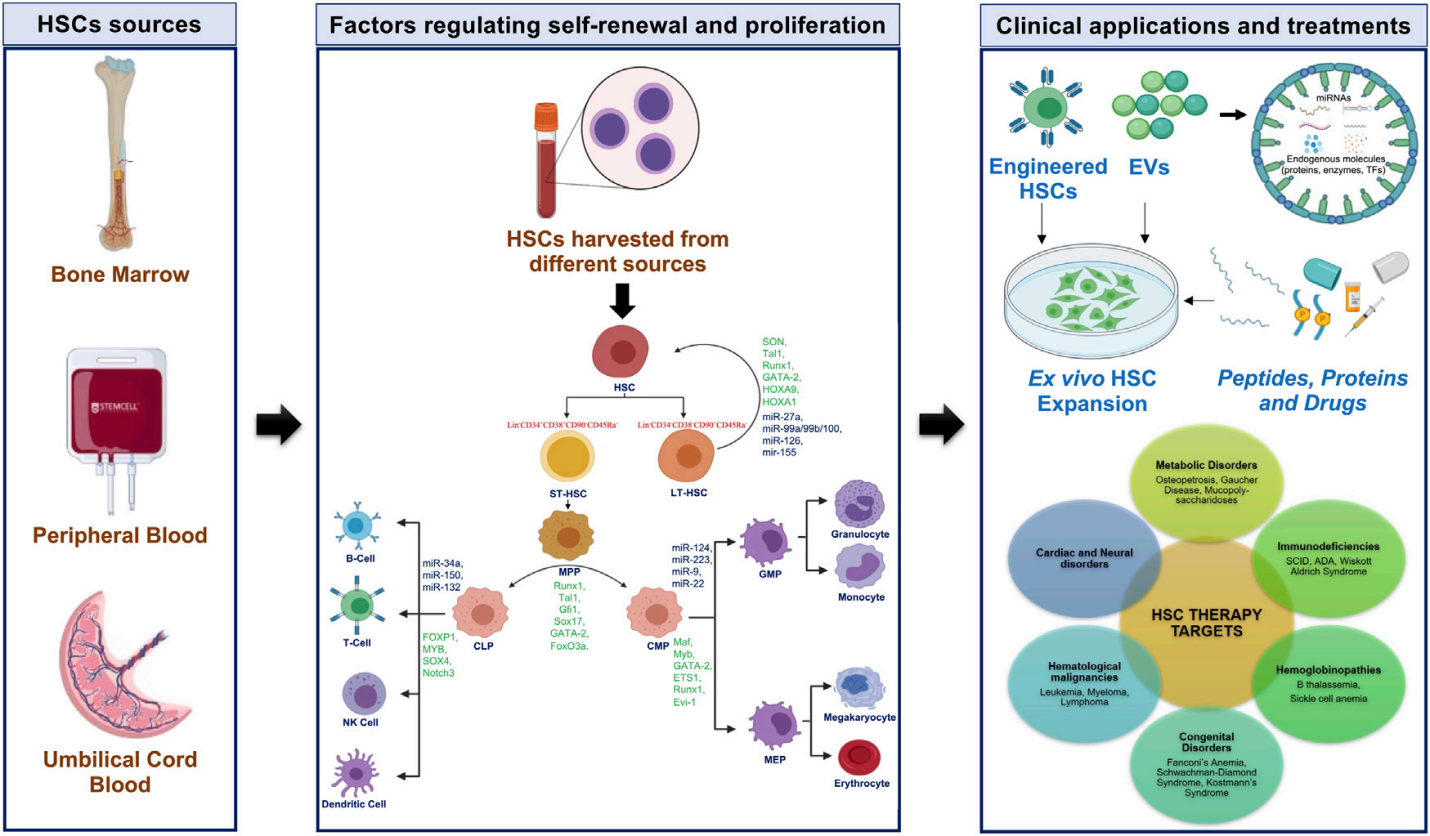
Human hematopoiesis with characteristic markers, TFs, and miRNAs with potential approaches for HSCs in clinical applications. CLP-common lymphoid progenitor; CMP-common myeloid progenitor; EVs-extracellular vesicles; GMP-granulocyte-macrophage progenitor; HSC-hematopoietic stem cell; LT-HSC-long-term HSC; MEP-megakaryocyte-erythrocyte progenitor; MPP-multipotent progenitor; NK-natural killer; ST-HSC-short-term HSC; TFs-transcription factors.

A better understanding of the transcriptional process regulating the self-renewal of HSCs is essential to enhance the shelf-life and potency of HSCs so that the frequency of HSCs isolation from donors and the invasiveness of the process can be minimized. This review summarizes the intrinsic factors that affect HSCs homeostasis and lineage commitment (Figure 1).

## 2 Hematopoiesis

*In vivo* evidence and colony-forming assays revealed two primary characteristics of HSCs-self-renewability and multipotency (Morrison et al., 1995; Orkin, 2000; Reya et al., 2001; Pazianos et al., 2003; Wilson et al., 2008; Foudi et al., 2009; Raghav et al., 2018a; Raghav et al., 2018b; Cheng et al., 2020; Grenier-Pleau and Abraham, 2021). The self-renewability of HSCs commands its top hierarchy in the classical lineage model of hematopoiesis.

The first evidence of the cell cycle dynamics of murine HSCs revealed that 99% of HSCs undergo cell division every 2 months, usually remaining in the G_0_ phase (Post and Clevers, 2019). Thereafter conducted *in vivo* studies revealed that dormant HSCs carry the highest self-renewal potential among all other blood cells (Wilson et al., 2008; Foudi et al., 2009). This quiescent HSCs subset arises through asymmetric division, retaining long-term self-renewal potential and producing an actively dividing progenitor subset that maintains the lineage commitment (Post and Clevers, 2019).

*Ex vivo* expansion of HSCs has established considerable heterogeneity within the HSCs pool due to asymmetric cell division (Post and Clevers, 2019; Wilkinson et al., 2019). This leads to the production of actively proliferating MPPs that differentiate step-wise into a restricted lineage based on the signals received from the niche, including the cytokines and TFs. This phenomenon was established through an immunophenotype-based tree-like hierarchy model given by Weissman’s group, which describes the classic HSCs harboring self-renewal property retained for more than three rounds of transplantation when delivered into recipients that can sustain hematopoiesis (Kondo et al., 1997; Morrison et al., 1997; Akashi et al., 2000; Manz et al., 2002; Cheng et al., 2020). Nevertheless, lack of equilibrium between the labeled HSCs and their progeny revealed that mature blood cells are replenished by a source of progenitor cells that differ from the HSCs in their cell cycle dynamics and possess higher self-renewability (Busch et al., 2015; Cheng et al., 2020).

In summary, symmetric or asymmetric cell divisions within the HSCs pyramid control self-renewability, egression from BM, and apoptosis. The proliferation rate of HSCs is almost twice that of the differentiation rate, establishing that a sub-population of HSCs maintains its pool by replacing the lost cells either through regression or cell death. Thus, symmetric cell division is more prevalent within the BM niche than asymmetric divisions (Cheng et al., 2020).

## 3 Classical Hierarchical Map

### 3.1 *In vivo* Fate Mapping

*In vivo* fate mapping enabled quantification of the net proliferation rate of the HSCs. The output is determined by the differentiation rate that balances the net proliferation as downstream components of the lineage model are modulated by the upstream components (Busch et al., 2015; Cheng et al., 2020). This sustains the self-renewal potential, the hallmark of hematopoiesis, and safeguards the HSCs lineage pool from the emergence of malignancies since the mutation rate is higher in the rapidly proliferating progenitor cells. The downstream components can be classified as two different populations based on the surface markers present. Hierarchy-wise, LT-HSCs differentiate into ST-HSCs, which further transform into MPPs, losing their self-renewability (Kondo et al., 1997; Morrison et al., 1997; Akashi et al., 2000; Yang et al., 2005; Cheng et al., 2020).

The MPPs subsequently bifurcate into CLPs and CMPs cell types, differentiating into the lymphoid and myeloid lineages, respectively (Yang et al., 2005; Cheng et al., 2020). The CLPs produce B, T, NK, and dendritic cells lineages, while CMPs differentiate into 1) bipotent GMPs, differentiating into granulocytes and monocytes; and 2) MEPs generating megakaryocytes and erythrocytes (Zhu and Emerson, 2002; Robb, 2007; Metcalf, 2008; Zhang and Lodish, 2008; Seita and Weissman, 2010).

Inducible genetic systems have been developed for label retention assays to gain more in-depth insight into the self-renewal and differentiation patterns of HSCs (Upadhaya et al., 2018). The existence of a dormant and rare HSCs subset was studied with the H2B-GFP expression model designed to regulate the CD34-tTA transgene system (Upadhaya et al., 2018). Herein, a small subpopulation was observed to retain the label stably even after 22 months. These cells had an excellent potency for repopulation in serial transplantation, while a large chunk of the population lost the label after four rounds of cell division (Bernitz et al., 2016). This revealed the presence of the quiescent HSCs population reserved even with progressing age. This subset of HSCs is also confirmed to have elevated retinoic acid (RA) signaling, as disclosed by G-protein coupled receptor family C group 5 member C (Gprc5c) labeling. Nevertheless, more studies are needed to confirm the effects of this signaling on HSCs self-renewal (Ghiaur et al., 2013; Upadhaya et al., 2018).

Fate mapping and physiological HSCs output are determined quantitatively and qualitatively by genetic labeling and barcoding (Emmons et al., 2017; Höfer and Rodewald, 2018). While the former labeling system is suitable for delineating the kinetics of self-renewal and differentiation rates of HSCs, the latter is more apt in probing physiological progenitor-product relationships (Emmons et al., 2017). Time-resolved fate mapping also confirmed the two kinds of the hematopoietic population: the dormant LT-HSCs and the actively dividing ST-HSCs (Lajtha et al., 1962; Trumpp et al., 2010; Emmons et al., 2017; Grinenko et al., 2018). Several *in vivo* fate mapping models have been established for an in-depth study of the same, an important one being the Tie2-Cre mouse model, wherein labeled Tie2^+^ cells are heritable at different stages of ontogeny (Gomez Perdiguero et al., 2015; Emmons et al., 2017). Tie2^+^ HSCs are placed at the top of the lineage chart, potent enough to generate all cell types downstream and maintained within the marrow throughout the mouse’s lifespan, confirming the self-renewal function (Foudi et al., 2009; Busch et al., 2015). Additionally, paired daughter assay revealed that symmetrically dividing Tie2^+^ cells sustain within the BM niche for a steady-state production of the HSCs (Cao and Ma, 2011).

In addition, HSCs display spleen colony-forming unit activity as determined by transplantation into lethally irradiated mice (Emmons et al., 2017). MPPs and associated progenitor cells are single cells that produce multiple progenies within the spleen to form secondary colonies during re-transplantation. It is possible due to the underlying self-renewal property of the cells (Ema et al., 2006; Emmons et al., 2016). In contrast, the barcoding experiments use unique transposon integration sites within fetal HSCs to delineate the precursor-progeny link within the adult HSCs pool (De Lisio et al., 2011). The erythroid progenitors and granulocytes share similar barcodes, implying a shared lineage of origin throughout fetal development (Bellows et al., 2011). Around two-thirds of these barcodes are also shared by CMPs, suggesting shared lineage. However, the remaining one-third population of erythroid progenitors and granulocytes with a different set of barcodes from the CMPs is plausibly due to limited self-renewal of CMPs within BM (Baker et al., 2011; Emmons et al., 2017).

### 3.2 Lineage Markers

Single-cell transplantation studies have established that multi-, oligo-, bi-, and uni-potent hematopoietic progenitors co-exist within the same niche (Cheng et al., 2020). Although the classical hierarchical model helps to visualize the hematopoietic process and marker-based isolation of individual cells, the cellular schematics defining the complexity of hematopoietic maturation at genetic and epigenetic levels are difficult to predict accurately (Xavier-Ferrucio and Krause, 2018).

Classic markers for mouse HSCs are Lin^−^, c-Kit^+^, Sca1^+^, Flk2^-^, CD34^−^ and Slamf1^+^, while human HSCs markers include Lin^−^, CD34^+^, CD38^+^ and CD90^+^. However, the expression levels for these markers vary between LT-HSCs and ST-HSCs (Huang et al., 2007). LT-HSCs are Slamf1^+^CD34^-^Flk2^−^CD34^−^, with levels of Lin^-^c-Kit^+^Sca1^+^ constant between the two population set in mice. In contrast, human LT-HSCs are CD34^−^CD90^+^ with the same expression levels in Lin^−^CD38^−^CD45Ra^−^ in ST-HSCs. The signaling lymphocyte activation molecule (SLAM) family markers, particularly CD150 and CD229, segregate mouse HSCs based on their potency. CD229^-^ LT-HSCs commit to myeloid lineage, while CD229^+^ HSCs commit to lymphoid lineage (Oguro et al., 2013). CD150^hi^ cells, on the other hand, have higher self-renewability than CD150^med^ HSCs and differentiate into myeloid lineage (Morita et al., 2010; Moignard et al., 2013).

Other markers identified through single-cell RNA sequencing (scRNA-seq) and single-cell assay for transposase-accessible chromatin using sequencing (scATAC-seq) revealed heterogeneity within the HSCs population (Moignard et al., 2013; Pietras et al., 2015; Wilson et al., 2015; Nestorowa et al., 2016; Buenrostro et al., 2018; Laurenti and Göttgens, 2018; Jacobsen and Nerlov, 2019). These cells are segregated into three main classes: LT-HSCs, ST-HSCs, and MPPs (Morita et al., 2010; Wilson et al., 2015; Post and Clevers, 2019). The ST-HSCs develop into MPPs, grouped into MPP1, MPP2, MPP3, and MPP4 populations as per their immunophenotypes and lineage bias. MPP1 resembles LT-HSCs with >4 months of multi-lineage reconstitution potency, while MPP2, MPP3, and MPP4 exhibit <1 month of the myeloid lineage potential (Manz et al., 2002; Wilson et al., 2008). Similarly, vWF^+^ platelet-primed HSCs also possess prolonged myeloid lineage bias and self-renewability (Sanjuan-Pla et al., 2013; Shin et al., 2014). The subsequent differentiated population sets have a constant expression pattern of markers Lin-c-Kit^+^ Sca1^-^ in mice and Lin^-^CD34^+^CD38^+^CD45Ra^-^ in humans. The difference in mouse and human CLPs is marked by Flk2^+^IL7Ra^+^CD27^+^ and CD10^+^ set of markers, respectively. Concurrently, mouse CMPs are identified as CD34^+^ FcgRlow and human CMPs as IL3Ralow. MEPs in mice express the markers set CD34^-^FcgR^-^, and IL3Ra^-^ in humans, whereas GMPs express CD34^−^FcgR^+^ and IL3Ra^+^ in mice and humans, respectively.

The transition within these three cell types is modulated by TFs such as Runx1, Tal1/SCL, Gfi1, Sall4, Etv6, Sox17, GATA-2, and FoxO3a. NFIL3 is expressed in CLPs and is required in innate lymphoid cell development within the BM (Sui et al., 1998). Similarly, T-cell factor 1 is essential for the differentiation of CLPs into natural killer T-cell progenitors. Another important TF is PU.1, functioning downstream of Runx1, controlling myeloid and B-cell differentiation (Heintz, 2001). PU.1 also regulates T-cell differentiation through Myb, T-cell factor 7, and GATA-3, checked by the Notch signaling (Post and Clevers, 2019). A comparison of the characteristic markers in mouse and human hematopoietic cell pools has been summarized in Table 2.

**TABLE 2:** Summary of characteristic markers of mouse and human hematopoietic cell population.

Cell type	Mouse markers	Human markers	PMID
Hematopoietic stem cells (HSCs)	Lin^−^c-Kit^+^Sca1^+^Flk2^−^CD34^−^Slamf1^+^	Lin^−^CD34^+^CD38^−^CD90^+^CD45RA^−^	20890962
Long-term HSCs (LT-HSCs)	Lin^−^c-Kit^+^Scal^+^Flt3^−^CD34^−^CD150^+^Thy1.1^+/low^	Lin^−^CD34^+^CD38^−^CD45RA^−^CD90^+^	31230184
	—	Lin^−^CD34^+^CD38^−^CD45RA^−^CD90^+^CD49f^+^	21737740
	—	CD34^+^CD45RA^−^ITGA3^+^EPCR^+^CD90^+^	31340144
Short-term HSCs (ST-HSCs)	Lin^−^c-Kit^+^Sca1^+^Flt3^−^ CD34^+^CD150^+^Thy1.1^+/low^Mac-1^low^	—	31230184
	—	Lin^−^CD34^+^CD38^−^CD45RA^−^CD90^−^CD49f^−^	21737740
	—	CD34^+^CD45RA^−^ITGA3^−^EPCR^+^CD90^+^	31340144
Multipotent progenitors (MPPs)	Lin^−^c-Kit^+^Sca1^+^Flk2^−^CD34^+^Slamf1^+^	Lin^−^CD34^+^CD38^−^CD90^−^CD45RA^−^	20890962
Common lymphoid progenitors (CLP)	Lin^−^Flk2^+^IL7RA^+^CD27^+^	Lin^−^CD34^+^CD38^+^CD10^+^	20890962
Common myeloid progenitor (CMP)	Lin^−^c-Kit^+^Sca1^−/low^CD34^+^FcgR^low^	Lin^−^CD34^+^CD38^+^IL3RA^low^CD45RA^−^	20890962
Megakaryocyte-erythrocyte progenitors (MEP)	Lin^−^c-Kit^+^Sca1^−^CD34^−^FcgR^−^	Lin^−^CD34^+^CD38^+^IL3Ra^−^CD45RA^−^	20890962
Granulocyte-macrophage progenitors (GMP)	Lin^−^c-Kit^+^Sca1^−^CD34^+^FcgR^+^	Lin^−^CD34^+^CD38^+^IL3Ra^+^CD45RA^−^	20890962

CD: cluster of differentiation; CLP: common lymphoid progenitors; CMP: common myeloid progenitors; EPCR: Endothelial Protein C Receptor; FcgR: Fragment of IgG receptor; Flk2: Fetal liver kinase 2; Flt3: FMS-like tyrosine kinase 3; GMP: Granulocyte-Macrophage Progenitors; HSC: hematopoietic stem cell; ITGA: Integrin Subunit α-1; Lin: Lineage; LT-HSC: Long-Term Hematopoietic Stem Cell; Mac-1: Macrophage-1, antigen; MEP: Megakaryocyte-Erythrocyte Progenitors; MPP: multipotent progenitors; Sca1: Stem Cell Antigen 1; Slamf1: Signalling lymphocytic activation molecule family member 1; ST-HSC: Short-Term Hematopoietic Stem Cell.

## 4 Factors for Hematopoietic Stem Cells Stemness and Self-Renewal

HSCs constitute <0.01% cells of the total BM population and maintain the hematopoietic system’s stability, owing to their self-renewal and differentiation potency (Shin et al., 2014; Upadhaya et al., 2018). The self-renewal property of LT-HSCs sustains the hematopoietic population, while the proliferation and differentiation capacity of ST-HSCs is empirical to produce multipotent progenitors (Emmons et al., 2017).

Extrinsic and intrinsic factors control the dynamic cellular organization within the hematopoietic system. Extrinsic factors include growth factors, hypoxia, and morphogens that activate signaling pathways, whereas intrinsic factors encompassing TFs, cell cycle regulators, epigenetic proteins, and miRNAs, as summarized in Table 1 (Manz et al., 2002; Beerman and Rossi, 2014; Zhao et al., 2014; Emmons et al., 2017).

The niche in which the cells reside is rich in TFs, miRNAs, and other mediators that provide a physical microenvironment for HSCs functioning. Quiescent HSCs reside within a perivascular niche, rich in endothelial cells and mesenchymal stem cells (MSCs) (Kunisaki et al., 2013; Morrison and Scadden, 2014; Scadden, 2014; Cabezas-Wallscheid and Trumpp, 2016; Pouzolles et al., 2016). The sinusoids localize approximately 85% of HSCs near Lepr^+^ and CXCL12^hi^ cells (Pouzolles et al., 2016). The hematopoietic progenitors migrate from the BM to the thymus settle near mKitL^+^ vascular endothelial cells, later shifting near mKitL^+^ cortical thymic epithelial cells (Pouzolles et al., 2016). This thymic niche is critical in supporting early progenitors’ differentiation into multiple hematopoietic lineages (Buono et al., 2016).

The BM is the primary site of hematopoiesis and this niche includes the endosteal and vascular niches (Tamma and Ribatti, 2017). The endosteal niche is localized in the inner bone shell of the trabecular and andocortical surfaces, primarily composed of osteoblasts, osteoclasts, macrophages, fibroblasts, endothelial cells and adipocytes. HSCs interact with osteoblasts via Tie2 and Ang-1 respectively, to maintain adhesion between the two cell types and thereby HSCs quiescence (Birbrair and Frenette, 2016). Immature osteoblasts with elevated Runx2 expression or those undergoing cell death lead to loss of hematopoiesis. Hence to compensate, immature osteoblasts upregulate CXCL12 surface expression to bind with CXCR4 in HSCs and support their maintenance. CXCL12 expression is however, negatively regulated by granulocyte-colony stimulating factor (G-CSF). Osteoblasts also negatively regulate HSCs pool *via* osteopontin surface expression.

The second type of niche is within the blood vessels called the vascular niche (Mirantes et al., 2014; Birbrair and Frenette, 2016). The vascular sinuses lined with endothelial cells are supported by the CXCL12-abundant reticular cells and MSCs, which form a reticular network and support HSCs formation, and maturation for megakaryocytic proliferation via the THPO/cMpl system. Other essential factors include FGF-4, VCAM-1 and VLA-4, which positively regulate platelet and endothelial cells maturation and proliferation. TGF-β negatively regulates HSCs proliferation, or rather promotes their quiescence, in addition to controlling c-Kit, IL6R, p21 and p57 expression levels. Additionally, hyaluronic acid produced by primitive hematopoietic cells enables HSCs migration to endosteal niche after transplantation.

### 4.1 Hematopoietic Regulators

Primarily, the TFs within the niche have a significant function in determining the fate of the HSCs. Several TFs have been identified for balancing the self-renewal and differentiation of HSCs (Zhu and Emerson, 2002). TFs such as Pbx-1 and Evi-1 maintain HSCs self-renewability, whereas SCL, Gfi1, PU.1, and FoxO maintain HSCs in the G_0_ phase. The Mef TF facilitates the quiescent HSCs transition from G_0_ to G_1_ phase (Huck et al., 2014). Other TFs that maintain the stemness of HSCs are SON, Tal1 or SCL, RUNX1, LMO2, GATA-2, HOXA9, and HOXA1, as summarized in Table 1 (Blume-Jensen et al., 1991; Shiohara et al., 1993; Rossi et al., 2007; Park et al., 2013; Wang and Ema, 2016; Bhatlekar et al., 2018).

#### 4.1.1 c-Myb

The Myb family of TFs is involved in cell cycle regulation and maintenance of genomic integrity, composed of three isoforms, a-Myb, b-Myb, and c-Myb. The latter two are majorly responsible for regulating hematopoietic functions (Baker et al., 2014). The b-Myb is a crucial factor in controlling cell fate that maintains the self-renewability of HSCs and progenitor cells, primarily in the G_0_-G_1_ phase. Depletion and microarray studies of b-Myb revealed the accumulation of HSCs population in S/G_2_ phases and decreased numbers of differentiated lymphoid, erythroid, and myeloid cells (Baker et al., 2014).

Similarly, c-Myb is a key self-renewal regulator in the early stages of HSCs development which acts in conjunction with PU.1 to enhance proliferation in the earlier set of HSCs, as reported in mice and human studies. The decrease in c-Myb expression leads to HSCs lineage commitment towards erythroid cells (Soza-Ried et al., 2010; Raghav and Gangenahalli, 2018). Therefore, it indicates that both these isoforms regulate cell fate in the early stages of the cell cycle.

#### 4.1.2 Ecotropic Viral Integration Site 1

Ecotropic viral integration site 1 (Evi1) is an oncogenic TF of the SET/PR domain protein family (Huck et al., 2014; Mirantes et al., 2014; Birbrair and Frenette, 2016). It has a developmental role in the hematopoietic system in the fetal and adult HSCs and progenitor cells. However, Evi1 deletion in mice embryos decreases HSCs numbers, whereas conditional deletion of Evi1 in adult mice disrupts HSCs development (Ficara et al., 2008; Kataoka et al., 2011). Nevertheless, Evi1 overexpression leads to myelodysplasia (Buonamici et al., 2004; Kataoka et al., 2011). Evi1 expression in LT-HSCs promotes stem cells self-renewal by interaction with downstream molecules like GATA-2, Pbx1, Runx1, and TGF-β (Yuasa et al., 2005; Senyuk et al., 2007; Sato et al., 2008; Goyama and Kurokawa, 2009; Shimabe et al., 2009). Forced and upregulated expression of Evi1 in HSCs prevents progenitor cells’ differentiation and enhances their expansion through inducing self-renewal in HSCs, independent of cell-cycle progression. Conversely, Evi1 heterozygosity causes loss of self-renewal in LT-HSCs and ST-HSCs, as reported in an Evi1^+/−^ mice model, which had diminished colony-forming efficiency (Kataoka et al., 2011).

#### 4.1.3 Forkhead O

The TFs belonging to Forkhead O (FoxO) family play a crucial role in diverse physiological processes, including apoptosis, regulating cell-cycle dynamics, and stress resistance (Lee and Castrillon, 2007). Four members of this family, FoxO1, FoxO3, FoxO4, and FoxO6, activate downstream PI3K-Akt signaling to sustain cell survival. Elevated FoxO levels maintain stemness within LT-HSCs by inhibiting cell cycle progression (Ludikhuize and Rodríguez Colman, 2020). FoxO1 and FoxO3 are required for metabolic regulation within LT-HSCs by glycolysis and fatty acid oxidation, keeping mitochondrial ROS levels to a sub minimal. However, mitochondrial activity increases during differentiation due to metabolic switch decreasing the FoxO levels and inducing MPP differentiation into various lineages (Ito et al., 2012; Ito et al., 2016). The loss of FoxO3 leads to defects in the long-term maintenance of the hematopoietic pool and LT-HSCs exhaustion because of absence or loss of repopulation ability (Miyamoto et al., 2007; Yalcin et al., 2008; Ito et al., 2016). Certain systemic factors also contribute to the functioning of FoxO proteins. Recent findings indicate that hyperglycemia and obesity disrupt the Akt-FoxO axis by upregulating oxidative stress that hinders the self-renewal of HSCs, decreasing the stem cell pool. Thus, specific hypoglycemic agents that can target PI3K/Akt pathway have a potential role in treating hematological deficiency disorders by upscaling the FoxO induced HSCs population (Govindarajah et al., 2020).

#### 4.1.4 GATA

The GATA family proteins, inclusive of the three isoforms GATA-1, GATA-2, and GATA-3, are essentially identified by two highly conserved zinc fingers of the C2H2 type that recognize the motif “WGATAR” (Martin and Orkin, 1990; Trainor et al., 1996; Gao et al., 2015). GATA-1 expression is required to develop lineage-committed hematopoietic cells such as CLPs and CMPs, which differentiate into megakaryocytes and erythrocytes, primarily through EPO signaling (Whyatt et al., 1997; Rylski et al., 2003). GATA-2 is required at the early stages of HSCs, subsequently replaced by GATA-1 once the differentiation process is initiated (Bresnick et al., 2010; Suzuki et al., 2013). GATA-2 loss of function mutation leads to dysfunctional adult human cord blood progenitors with self-renewal potential (Menendez-Gonzalez et al., 2019). Conversely, LT-HSCs are enriched with GATA-3, whose activation has correlated signaling through the MAPK-p38a pathway (Yoshida and Georgopoulos, 2013). The loss of GATA-3 in steady-state quiescent HSCs does not affect its self-renewal or proliferation (Yoshida and Georgopoulos, 2013). However, under stress-activated p38a conditions, GATA-3 deletion restrains the self-renewal of LT-HSCs by balancing proliferation and differentiation without affecting the cell cycle (Frelin et al., 2013; Yoshida and Georgopoulos, 2013).

#### 4.1.5 Growth Factor Independence

Growth factor independence 1 (Gfi1), a transcriptional repressor composed of Snail/Gfi1 (SNAG) domain and six zinc-finger motifs, is a part of the oncogenic complementation system as Bmi-1 (Moraes et al., 2002; Lecellier and Voinnet, 2004; Cellot and Sauvageau, 2005). The most studied function of Gfi1 is IL-2 dependent T-cell proliferation, in conjunction with Pim-1 and Myc TFs, and IL-6/STAT3-mediated proliferation in case of antigenic stimulation (Schmidt et al., 1998). Gfi1 depletion studies in adult HSCs revealed decreased long-term reconstitution capacity in a cell-autonomous manner. However, no significant change was observed in MEPs, suggesting their primary role in maintaining stemness and proliferation in stem cells and progenitors (Zeng et al., 2004). This function is triggered by the transactivation of GATA-2 and RUNX1 TFs by associating Gfi1 with enhancer Mysm1 (Wang et al., 2013). However, the effect of this factor on apoptosis or cell homing is yet to be studied.

#### 4.1.6 Homeobox

The homeobox (HOX) genes are a family of highly conserved homeodomain-containing TFs that specify cell type identity in the early stages of development. These can be categorized into four separate clusters- HOXA, HOXB, HOXC, and HOXD, located on different chromosomes. The expression of HOX proteins follows a specific pattern, with the HOXA gene expressed in myeloid cells, HOXB in erythroid cells, and HOXC in lymphoid cells (Alharbi et al., 2013). HOX 1-6 genes are usually expressed within a HOX cluster in stem and progenitor cells. In contrast, the genes in order of their arrangement on the chromosome are expressed in lineage-committed cells (Sauvageau et al., 1994; Alharbi et al., 2013). Specific HOX genes have been reported to enhance stem cell and progenitor populations by inhibiting differentiation. Overexpression of HOXC4 stimulates early myeloid and erythroid progenitors to proliferate, while HOXA9, HOXB4, and HOXB6 maintain stem cell pools and support proliferation (Kroon et al., 1998; Thorsteinsdottir et al., 2002). However, these are downregulated at the time of differentiation, sustaining the regenerative potential of the LT-HSCs and ST-HSCs (Sauvageau et al., 1994; Pineault et al., 2002). STAT3 is functionally similar to HOXB4, yet they do not synergize and show independent effects (Hong et al., 2014). HOXB6 is an essential TF to maintain the self-renewal potential of the HSCs, and any mutations in this gene can end up in AML (Kappen, 2000; Fischbach et al., 2005; Vainshtein et al., 2015).

#### 4.1.7 Mef

Mef or ELF4 is a member of the E26 transformation-specific (ETS) family of winged helix-turn-helix TFs and primarily acts on LT-HSCs (Lacorazza et al., 2006; Suico et al., 2017; Wilkinson et al., 2020). Its expression is dramatically reduced in cases of AML, implying its tumor-suppressive role (Fukushima et al., 2003). HSCs display enhanced levels of reconstitution in serial transplantation in the absence of Mef. On the contrary, Mef deficient HSCs do not show positive repopulation in primary transplantation, suggesting its main function in LT-HSCs self-renewal (Suico et al., 2017). It maintains quiescency within HSCs associated with p53, acting downstream of Gfi-1 and Necdin (Liu et al., 2009).

#### 4.1.8 Pbx-1

Pbx-1 is a TALE class homeodomain TF regulating critical embryonic processes such as organogenesis and hematopoiesis (DiMartino et al., 2001; Kim et al., 2002; Baker et al., 2014). The proto-oncogene Pbx1 has higher expression in LT-HSCs than in ST-HSCs and MPPs (Forsberg et al., 2005; Kiel et al., 2005). Pbx1 deficiency causes downregulated self-renewal and proliferation activity of HSCs and impaired long-term engraftment. It is known to exhibit this attribute *via* TGF-β and MAPK signaling (Baker et al., 2014). Pbx1 forms a transcriptional complex with Prep, acting on TGF-β and regulating the expression of p21 and p57 genes to maintain HSCs quiescence (Bromleigh and Freedman, 2000; Umemoto et al., 2005). Lack of Pbx1 has been associated with severe fetal anemia and disruption in lymphoid differentiation since the absence of Pbx1 leads to premature expression of ST-HSCs genes within LT-HSCs (Baker et al., 2014).

#### 4.1.9 PU.1

The PU.1 is another member of the ETS family of TFs, encoded by the proto-oncogene SPI1 (Tothova et al., 2007; Wilkinson et al., 2020). The expression of PU.1 is primarily linked to the differentiation and maturation of lineage-committed cells, including lymphoid, myeloid, and macrophage lineages (Dakic et al., 2005; Chang et al., 2010). Additionally, it maintains the stem cell pool in the hematopoietic system (Staber et al., 2013). Loss of functional mutation in PU.1 leads to the development of AML in adults and results in embryonic lethality (Scott et al., 1994; McKercher et al., 1996; Mueller et al., 2002; Iwasaki et al., 2005). It causes a maturation arrest in the transition of HSCs to CLP and CMP phenotypes, hence establishing PU.1 requirement for the competitive self-renewal of HSCs. However, a low level of PU.1 expression is required for HSCs maintenance, whereas an increased level causes myeloid differentiation (Iwasaki et al., 2005; Raghav and Gangenahalli, 2021). The exact mechanism of PU.1 is still under speculation; however, a recent study reported the role of non-canonical Wnt signaling in HSCs self-renewal and myeloid lineage differentiation (Welner et al., 2020).

#### 4.1.10 Runt-Related Transcription Factor 1

Runt-related transcription factor 1 (RUNX1) is an essential TF functioning at multiple hematopoiesis stages, including definitive HSCs formation and differentiation into granulocytes, B-cell, and megakaryocytes (Guo et al., 2012; Dowdy et al., 2013; Ichikawa et al., 2013; Tober et al., 2013; Gerritsen et al., 2019). Structurally, RUNX proteins have a 128 amino acid long DNA-binding domain (at N-terminus) and a conserved five amino acid motif, “VWRPY” (at the C-terminus). The N-terminal binds to a consensus DNA sequence “TGTGGT” or “TGCGGT” and regulates hematopoiesis by modulating the expression levels of the PF4 gene, IL-3 GM-CSF cytokines, and M-CSFR cytokine receptors (Harada et al., 2013; Gerritsen et al., 2019). It plays a significant role in embryonic stage hematopoiesis by promoting the development of vascular endothelial cells. However, in adult HSCs, RUNX1 expression leads to stem cell exhaustion, indicating that its deletion would affect hematopoietic stem/progenitor cells (Osato, 2004; Haferlach et al., 2016).

Mutations in the RUNX1 gene correlate with the occurrence of AML and myelodysplasia, suggesting that this TF has a tumor-suppressive function. The conserved RUNX family has three isoforms, RUNX1, RUNX2, and RUNX3. Molecules regulating RUNX1 activity include ERK, HIPK2, cyclin-dependent kinases, or methylators like PRMT1 (Preudhomme et al., 2000; Gerritsen et al., 2019). RUNX1 was reported to function as a cytoplasmic regulator of NF-kB signaling, interacting with the IkB kinase complex. The NF-kB inhibitor BMS-345541 inhibits the proliferation of leukemia cells containing RUNX1 mutation, suggesting a potential role of NF-κb inhibitors in targeting RUNX1-related leukemia (Gerritsen et al., 2019).

#### 4.1.11 Stem Cell Leukemia

SCL is a key factor required at different stages of HSCs development in SCL. Transcription of this gene is reportedly the highest in quiescent HSCs at the G_0_ phase compared to more actively proliferating HSCs in the G_1_/S/G_2_ phase (Lacombe et al., 2010; Rojas-Sutterlin et al., 2014; Haferlach et al., 2016). It regulates a smooth G_0_ to G_1_ transition of LT-HSCs by acting on its partners E47, GATA-2, and LDB1 (Rojas-Sutterlin and Hoang, 2013). SCL acts upstream of c-Kit to sustain self-renewal. However, the entire SCL-c-Kit axis is yet to be delineated (Rojas-Sutterlin and Hoang, 2013; Rojas-Sutterlin et al., 2014). Heterozygous expression of SCL exhibited repopulation efficiency limited to only 4 months of post-transplantation and impaired repopulation capacity after second transplantation (Lacombe et al., 2010). This study corroborated another study wherein, enforced SCL expression enhanced regenerative potential in secondary transplantations (Reynaud et al., 2005). SCL deletion can be compensated by Lyl1 expression, but not vice-versa (Fujiwara et al., 2004; Chan et al., 2007). In addition to its role in development, SCL controls the cellular transition from a proliferating stage to erythroid progenitors’ formation (Lacombe et al., 2010). This activity is exhibited by the upregulation of Gfi1b and Cdkn1a gene expression. The upregulated expression of cell cycle regulators Cdkn1a and Id1 maintain quiescency and mitotic index in LT-HSCs (Lacombe et al., 2010).

#### 4.1.12 c-Kit

c-Kit maintains a balance between self-renewal and differentiation among HSCs via the c-Kit/SCF axis (Mirantes et al., 2014; Birbrair and Frenette, 2016). The c-Kit^lo^ population stays higher in the hierarchy than c-Kit^hi^, and the transition from c-Kit^lo^ to c-Kit^hi^ is mediated by c-Cbl (Shiohara et al., 1993; Huck et al., 2014). It is observed that c-Kit^lo^ HSCs exhibit long-term reconstitution with enhanced self-renewal as compared to c-Kit^hi^. The cells expressing c-Kit^hi^ also show increased GATA-1 and decreased HOXB4 expression levels, making this population more biased towards megakaryocytic differentiation (Huck et al., 2014). SCF cytokine acts as a ligand for the c-Kit receptor to maintain self-renewal and proliferation of HSCs (Rossi et al., 2007). SCF in synergism with GM-CSF, IL-3, and EPO activates c-Kit intrinsic tyrosine kinase activity that signals the PI3K/MAPK pathway to switch to proliferation (Park et al., 2013; Raghav and Gangenahalli, 2018). Thus, for *ex vivo* expansion of HSCs, ablating c-Kit levels in conjunction with SCF might be a suitable strategy by upregulating the self-renewal levels while acting in synergy with GATA-1 that steer lineage-specific differentiation.

### 4.2 miRNAs

Some miRNAs have a functional role in self-renewal of HSCs viz. miR-27a, miR-99a/99b/100, miR-126, mir-155 and miR-33/142a-3p (Gekas and Graf, 2013). miRNAs also play a significant role in HSCs differentiation into a particular lineage. miRNAs responsible for controlling differentiation to myeloid lineage are miR-124, miR-15a/16, miR-486-3p, miR-223, mi144/451, miR-9, and miR-22. Besides, miR-34a, miR-150, and miR-132 control differentiation of HSCs into lymphoid lineage (Gekas and Graf, 2013; Xavier-Ferrucio and Krause, 2018).

The miR-99 family control HSCs quiescency and self-renewal by regulating HOXA1 levels, while miR-126 keeps HOXA9 levels under check to suppress leukemogenesis (Gekas and Graf, 2013; Bhatlekar et al., 2018). The tumor suppressor protein p53 is another TF that controls HSCs quiescency. The miR-33 and miR-142-3p are essential regulators of p53. These miRNAs are associated with decreased cell proliferation and increased apoptosis to check any leukemia incidence (Vainshtein et al., 2015; Foroutan, 2017).

The signaling pathways, Notch, Wingless-type (Wnt), Sonic hedgehog (Shh), and Smad, control the LT-HSCs population. Moreover, the IL-6 and IL-11 receptor, gp130 protein, and c-Kit play an essential role in HSCs self-renewal (Velasco-Hernandez et al., 2016; Hashimoto et al., 2021). Intracellular adaptor molecule, Lnk negatively regulates thrombopoietin (TPO), SCF, erythropoietin (EPO), IL-3, and IL-7 signaling pathways, decreasing HSCs self-renewal potency. Nevertheless, cytokines, SCF, and TPO are the two most important positive regulators of HSCs. Reportedly, the forced expression of β-catenin upregulates the expression of Notch1 and HOXB4 genes, enhancing HSCs self-renewal (Emmons et al., 2017; Xavier-Ferrucio and Krause, 2018).

### 4.3 Epigenetics

Another approach to regulate the HSCs expansion is *via* a better understanding of the epigenetic regulators. A crucial epigenetic regulator is MLLT3 or AF9, which in conjugation with DOT1L, acts as histone reader to methylate H3K79 and maintains the self-renewability of HSCs. MLLT3 levels decrease in HSCs cultured *in vitro;* thus, stabilizing MLLT3 levels in culture and causing a 12-fold increase in expansion of transplantable HSCs (Calvanese et al., 2019). Similarly, bromodomain PHD finger transcription factor (BPTF), an essential component of the nucleosome remodeling factor (NURF) chromatin-remodeling complex, maintains both LT-HSCs and ST-HSCs population. BPTF knockout leads to defects like anemia by suppressing Meis1, Pbx1, Mn1, and Lmo2 genes required for self-renewal (Xu et al., 2018).

Nevertheless, C/EBPα regulates multiple cell fates like directed myeloid differentiation in HSCs, maintaining self-renewal and quiescent state in LT-HSCs acting as an apoptotic inhibitor. Deleting the C/EBPα gene leads to the exhaustion of the HSCs population by recruiting epigenetic writers and erasers, a mechanism still elusive (Hasemann et al., 2014). The TF hepatic leukemia factor (HLF) also regulates HSCs quiescence and leukemogenesis. RNA-Seq data of HLF deficient mice revealed 550 differentially expressed genes, including cell cycle control genes’ upregulation. Gene ontology (GO) and gene set enrichment analysis (GSEA) study further elucidated that these upregulated genes primarily function for cell cycle regulation, DNA replication, and cellular stress response. The Chip-Seq data revealed that HLF also downregulates certain genes like GFI1 and IRF2, highlighting its dual role in promoting and inhibiting HSCs self-renewal genes (Komorowska et al., 2017).

Additionally, HSCs require a constitutive level of DNA methylation to sustain self-renewal status. The key players in this methylation include DNMT3a and DNMT3b, MLL1 and MLL5, Ezh2, Kdm2b, Sirt1, and CHD4/NuRD complex (Komorowska et al., 2017). TCF7 is an HMG box protein that regulates self-renewal in HSCs. RNA-Seq and GSEA data revealed that TCF7 is suppressed when HSCs differentiate from CD44^+^ to CD44^−^ cells and thus play a dual role in promoting self-renewal and inhibiting differentiation promoting genes (Wu et al., 2012). On a similar note, DNA acetylation is also very crucial in HSCs population maintenance, as noted by DNA acetylatases KAT6A, HDAC1, and HDAC2 activity (Jiang et al., 2019). In addition, acetylation is a crucial regulator as lysine acetyltransferases (KATs) such as p300, CBP and HBO1, alter the chromatin structure for gene activation. CBP-p300, which belongs to the KAT family, regulates both differentiation and self-renewal by binding to c-Myb KIX domain and regulating its gene expression (Rodrigues et al., 2021). MOF is a different KAT that regulates erythropoiesis by modulating H4K16ac. This is counterbalanced by histone deacetylases (HDAC) like SIRT1, HDAC1 and HDAC2. Additionally, Mi2β of the NuRD complex is an SNF2-like ATPase required for self-renewal.

### 4.4 Negative Regulators

Within the hematopoietic niche, many growth factors and signals maintain HSCs quiescence and self-renewal stage. However, some other prevalent factors act as negative regulators, such as the immune signals that stimulate HSCs exit from quiescence and differentiation most commonly into immune regulatory cells (Caiado et al., 2021). Common trigger factors for inciting this HSCs differentiation and immune response are microbiota, bacterial or viral infection, carcinogens, inflammatory diseases and aging. For instance, the anti-viral immune response causes IFN-α production which acts as a negative HSCs regulator and triggers its differentiation into the myeloid lineage. However, bacterial response produces IFN-γ by T and NK cells which also promotes granulopoiesis (Zhang et al., 2022). Other pro-inflammatory cytokines mediate functional effects on HSCs, such as TNF-α binding with HSCs to induce apoptosis. In fact, excessive TNF-α production is linked with BM failure and myelodysplastic syndrome (MDS). Additionally, release of pathogen-associated molecular patterns (PAMPs) increases in cases of bacterial infection, which bind to the HSCs receptors like toll-like receptors (TLRs) and act as niche regulators (Barman and Goodridge, 2022). For example, human cord blood HSCs produce pro-inflammatory cytokine IL-8 *via* MAPK/AP-1 signalling when CpG DNA from microbes activate TLR9 present on the HSCs surface. Human BM HSCs express TLR4 and TLR7/8 instead which when activated lead to differentiation into monocytes.

Moreover, PU.1 and GATA-1 TFs are maintained in a balanced ratio to regulate the HSCs, yet their respective increase leads to differentiation into myeloid and erythroid lineages (Li, 2011). TNF-α furthermore increases PU.1 production, driving myelopoiesis. These regulators can be divided based on their extrinsic or intrinsic presence. For example, the hedgehog (Hh) signalling pathway is an extrinsic negative regulator, while factors such as MEF/EFL4, Lnk and c-Myc are intrinsic negative regulators of hematopoiesis. The differentiated niche cells play a significant role in regulating HSCs differentiation (King and Goodell, 2011). CXC-chemokine ligand 12 (CXCL12), secreted by osteoblasts in the bone marrow, binds to CXCR4 receptor on HSCs to retain them in the marrow. However, upregulated G-CSF levels during infections, suppress the CXCL12 secretion to mobilize HSCs out of the marrow and differentiate depending on the niche signal.

### 4.5 Oxidative Stress and Autophagy

The BM multifarious niche factors play a central role in the homeostasis of the hematopoietic process (Hu et al., 2019). Given that HSCs are placed at the top of the hierarchy, their self-renewal and differentiation potential give rise to various peripheral blood cells *via* hematopoietic progenitors (Miyamoto et al., 2007; Laurenti and Göttgens, 2018). One of the metabolic niche factors modulating this process is oxidative stress, governed by reactive oxygen species (ROS) (Ito et al., 2007; Suda et al., 2011). ROS formed as the byproducts of signal transduction and energy metabolism during hematopoietic regulation and can be beneficial and harmful to the cells depending on their fluctuating intracellular levels (Hu et al., 2019). However, quiescent HSCs require ROS levels to maintain their stemness, elevated ROS levels within the HSCs promote migration, and differentiation *via* the p190-B RhoGAP-ROS-TGF-β-p38MAPK signaling (Ito et al., 2007; Suda et al., 2011; Ludin et al., 2014; Shinohara et al., 2014; Hinge et al., 2017; Golan et al., 2018; Hu et al., 2019). However, an abnormally high ROS concentration may affect HSCs self-renewability, impair proliferative capacity, and cause DNA damage and cell cycle arrest. The two most recognized endogenous sources of intracellular ROS production are mitochondria and membrane NADPH oxidase (NOX) (Bigarella et al., 2014; Willems et al., 2015). Besides, ionizing radiation (IR) is the most widely accepted exogenous source of ROS production in the body (Li et al., 2016a). BM is the most susceptible tissue to IR. IR causes acute and long-term BM suppression, eventually leading to severe hematological symptoms. This suppression elevates ROS levels within the BM that impair HSCs functioning, induce senescence and stem cell exhaustion. This process is mediated by the p38MAPK-p16ink4a pathway (Shao et al., 2014).

The aggravated ROS concentration can be alleviated by antioxidants, such as Nrf2, a master TF regulating other antioxidant enzymes such as catalase, SOD, GPX1, and thioredoxin. Commercially available antioxidant supplements such as resveratrol, metformin, chlorophyllin, and methoxytryptamine-α-lipoic acid serve the same function, thus mitigating IR induced hematopoietic abnormalities (Zhang et al., 2013; Suryavanshi et al., 2015; Xu et al., 2015; Li et al., 2016b). Nrf2 function as an essential redox regulator of HSCs [187]. For example, in mice, Nrf2^−/−^ HSCs exhibited significant proliferation, phenotypically similar to FoxO deficient HSCs. Hence, it was speculated that Nrf2 and FoxO work parallelly downstream of PI3K/Akt pathway to control HSCs proliferation (Tsai et al., 2013). According to a recent study, LT-HSCs differentiation and cell cycle entry is modulated by Nrf2 and further maintained by Keap1, compromising the HSCs quiescence status (Murakami et al., 2017).

Similarly, this factor is a global regulator of other stem cell types as well. It contributes towards survival, quiescence, and stemness in embryonic stem cells (ESCs), MSCs, intestinal stem cells (in *Drosophila*)*,* and stress resistance in cancer stem cells (CSCs) (Paul et al., 2014; Ryoo et al., 2016; Ge et al., 2017).

Another mechanism that keeps oxidative stress under control in the BM niche and HSCs functioning intact is autophagy. Broadly, autophagy regulates remodeling during terminal differentiation, delays senescence, and balances the self-renewal and quiescent attributes of the hematopoietic population. These outcomes are modulated via the ATG genes and mTOR/AMPK signaling pathway (Mizushima and Levine, 2010). Among a total of 40 ATG genes, those identified to play a prominent role in stem cell quiescence are PINK1, ULK2, ATG8 homologs MAP1LC3A, GABARAPL1, and FoxO3 (Cheung and Rando, 2013; Murakami et al., 2017). FoxO3A, for instance, induces autophagy in HSCs through ATG4B, BNIP3, and MAP1LC3B, to protect the cells from metabolic stress during starvation (Warr et al., 2013; Riffelmacher and Simon, 2017). The highest autophagy rate is seen in HSCs, decreasing their further differentiation into progenitors (Warr et al., 2013; Watson et al., 2015; Riffelmacher and Simon, 2017). The HSCs differentiation is also defined by the metabolic switch from anaerobic glycolysis to mitochondrial oxidative phosphorylation (OXPHOS). This switching occurs because of the mTOR pathway’s inhibition, which induces autophagy and suppresses OXPHOS, contributing to HSCs quiescence (Gan et al., 2010; Gurumurthy et al., 2010; Butler et al., 2018). The HSCs labeled with Tie2-GFP transgenic reporter are capable of serial transplantation, rendering about 5% of the cells Tie2-GFP^+^ (Upadhaya et al., 2018). These positively labeled cells undergo proliferator-activated receptor (PPAR) signaling and mitophagy (Watson et al., 2015). The *in vivo* paired daughter cell assays proved that the Tie2-GFP^+^ cells had measurable self-renewal and differentiation capacity but decreased on Parkin knock-down, a key molecule in mitophagy. This established the link between mitophagy and self-renewal within the HSCs (Motoike et al., 2000).

Autophagy also controls the dynamics of the differentiated immune cells. For example, plasma cells have higher autophagy levels than CD19^+^ B cells. This is controlled through ER homeostasis via reticulophagy, thereby limiting the ROS concentration (Pengo et al., 2013). B- and T-cell differentiation into memory cells is also associated with increased autophagy rate, as detected by the quantification of LC3-II, ATG5, and ATG7 genes (Chen et al., 2014). Recent experiments suggest that BNIP3- and BNIP3L-dependent mitophagy is essential in natural killer (NK) cells to sustain self-renewal and cytoplasmic homeostasis by downplaying ROS levels (Salio et al., 2014).

## 5 Regulating Hematopoietic Stem Cells for Clinical Use

### 5.1 Engineered Hematopoietic Stem Cells

Cell engineering has shown insights into directed differentiation and TF-regulated approaches to design patient-specific HSCs. The sustained self-renewal and controlled differentiation to produce a functional and stable hematopoietic system is required for achieving therapy-grade engineered HSCs (Rowe et al., 2016). Heterochronic genes impact the HSCs in a stage-specific manner to regulate the hematopoietic system’s development, observed in transplantation assays (Rowe et al., 2016). The primary objective of TF-mediated blood engineering is to evade these heterochronic barriers’ complexity to reach adult HSCs phenotypes. The LIN28 and let-7 family of miRNA can be fine-tuned in engineered HSCs to enhance self-renewal potential for therapeutic applications (Rowe et al., 2016). However, using small molecules instead of the growth factors and cytokines might be a more economical and impactful alternative (MacRae and Peterson, 2015). For *in vivo* imaging, zebrafish has come up as a good model, giving better insights into the early developmental stages of hematopoiesis. Also, this has facilitated the identification of crucial factors required in these development stages. For instance, chromatin modifiers such as Bmi-1 maintain the quiescency, and Ezh1 drives HSCs to senescence in its absence (Park et al., 2003; Hidalgo et al., 2012). The gene therapy has been proven clinically efficient in targeting X-linked severe combined immune deficiency (X-SCID), adenine deaminase deficiency SCID (ADA-SCID), and chronic granulomatous disease (CGD). However, this technique also runs the risk of mutagenesis by specific proto-oncogene activation. Therefore, engineered HSCs would be a promising strategy for better clinical outcomes, a more in-depth understanding of the hematopoietic system is required to build functionally stable HSCs. Such HSCs are expected to have transfusion independence and acquire a stable immune system with diverse blood cells.

### 5.2 *Ex vivo* Hematopoietic Stem Cells Expansion

*In vivo* HSCs expansion is balanced by self-renewal and differentiation through various TFs and growth factors. However, *in vivo* persistence of HSCs within transplanted patients and their *ex vivo* expansion have been a significant obstacle in clinical practice. Thus, simulating a similar environment in *ex vivo* cultures by identifying the optimal growth factors such as thrombopoietin, SCF, Flt3 ligand, IL-6, and G-CSF is a pragmatic approach to sustain the isolated HSCs colonies outside the body (Papa et al., 2020). Several strategies have been devised using umbilical cord blood-derived HSCs (UCB-CD34^+^) for their *ex vivo* expansion (Table 3) (Papa et al., 2020).

**TABLE 3:** Chemical molecules used in *ex vivo* HSCs expansion.

Molecules	Functions	PMID
5-HT	Decreases caspase levels and inhibits HSCs apoptosis	17446559
AMD3100	Mobilizes HSCs circulation into peripheral circulation by inhibiting CXCR4 activity	24012368
BIO5192	Mobilizes HSCs circulation into peripheral circulation by inhibiting VLA-4 activity	24012368, 29987969
CASIN	Stimulates renewal of aged HSCs any inhibiting Cdo42	24012368
DEAB	Inhibits HSCs differentiation via ALDH suppression and downregulating HOXB4 and cEBPε levels	16857736, 29987969
Diprotin A	Stimulates HSCs for homing and renewal by inhibiting DPP4	24012368
dmPGE2	Promotes cell homing *in vivo* through the PGE pathway and enhances HSCs engraftment	19324903, 17581586, 24012368, 29987969
Garcinol	HAT inhibitor that stimulates HSCs renewal	21931675, 29987969
Nicotinamide	Enhances *in vivo* cell homing by SIRT1 inhibition and decreasing p21 expression	22198152, 29987969
NR-101	Enhances self-renewal by HIF-1α and VEGF stabilization	19744539, 29987969
OAC1	Expands early MPPs by upregulating HOXB4 and Oct4 expression	29987969
P18IN003/011	Enhances HSCs self-renewal via p18INK4c inhibition	25692908, 29987969
Rapamycin and CHIR99021	Stimulates HSCs proliferation *ex vivo* by mTOR inhibition and activating Wnt pathway	23142822, 29987969
SB203580	Triggers HSCs proliferation by inhibiting apoptosis via p38 MAPK	29987969
SR-1	AhR antagonist required for self-renewal	20688981
TEPA	Supports self-renewal by decreasing acetylation levels of histones	16219531
TSA	DNA methyltransferase inhibitor that promotes self-renewal	25363624
UM171	Enhances self-renewal and quiescence in LT-HSCs	25237102
UM729	Enhances HSCs self-renewal	25237102
VA	HDAC inhibitor promotes reprogramming by replacing Klf4 and also inhibits HSCs differentiation	18568017, 18509334, 15735039
zVADfmk	Blocks apoptosis by suppressing caspase activity and upregulating Bcl-2 and Notch1 expression	20808921, 29987969

5-HT: 5-Hydroxytryptamine; AMD3100: AnorMeD-3100; DEAB: diethyl azodicarboxylate; dmPGE2: 16,16-Dimethylprostaglandin E2; SR1: Stem-Reginin; TEPA: tetraethylenepentamine; TSA: trichostatin; VA: valproic acid.

One method to go about it is by Notch signalling activation within HSCs. Treating HSCs with an engineered Delta-like ligand like DELTA1^ext−IgG^ (DXI) under normoxic conditions activates the Notch pathway in ST-HSCs by triggering the transcriptional activity of HES and HEY genes responsible for self-renewal (Araki et al., 2021). However, this engraftment did not sustain for long since it was ineffective on the LT-HSCs. Therefore, to counter the oxidative stress implicated on the endoplasmic reticulum under normoxic conditions, HSCs expanded *ex vivo* with exogenously provided DXI in hypoxia manifested an almost five fold increase in LT-HSCs compared to the untreated cells, offering a clinical potential for the long term HSCs sustenance *ex vivo* (Araki et al., 2021). The hypoxic condition also supports the stability of HIF-2α heterodimerization with HIF-1β. Since unbound HIF-1β under normoxia binds to aryl hydrocarbon receptor (AhR) that triggers transcription of cellular differentiation genes CYP1A1 and CYP1B1, confirming the crucial role of AhR pathway in self-renewal (Araki et al., 2021). AhR antagonists like Stem-Reginin1 (SR1) can increase the HSCs numbers by 50-fold and establish long-term engraftment in immunodeficient mice (Boitano et al., 2010). A phase I/II clinical trial was conducted wherein SR1 treatment to UCB-CD34^+^ cells demonstrated rapid platelet and neutrophil recovery. However, the mechanism of action is yet to be explored (Wagner et al., 2016; Zgurskaya and Rybenkov, 2020). A pyrimidoindole derivative, UM171, enhances primitive HSCs pool efficiently than SR1, independent of the pathway (Fares et al., 2014). This was characterized by the expression of endothelial protein C receptor (EPCR) over CD34^+^CD90^+^ cells as endothelial protein C receptor (EPCR) activates protease-activated receptor-1 signaling and displays a transcriptome similar to that of freshly isolated HSCs (Fares et al., 2014). Epigenetic modifiers, valproic acid (VA), and deacetylase inhibitor functionally reprogram UCB-CD34^+^ to acquire transcriptomic and mitochondrial profiles resembling that of primary HSCs (Fares et al., 2014; Papa et al., 2020). The high expression levels of CD90 and EPCR elevates the self-renewal and quiescence of HSCs. Similarly, p53 activity is controlled by suppressing intracellular Ca^2+^ levels and causes a slower cycling rate and higher self-renewal potential (Umemoto et al., 2018). Other essential factors considered for *ex vivo* HSCs expansion include mitochondrial activity. HSCs depend on glycolysis for energy production at the quiescent stage of self-renewal, displaying minimal mitochondrial activity (Jang and Sharkis, 2007; Romero-Moya et al., 2013; Warr and Passegué, 2013; Vannini et al., 2016). Low mitochondrial activity is linked to lower mitochondrial mass, membrane potential, and lesser ROS production. Following myeloablation, connexin-43 also mediates ROS transfer from HSCs to MSCs, keeping oxidative stress in check (Taniguchi Ishikawa et al., 2012; Qian et al., 2016). This tightly synchronized process is coordinated by mitophagy and autophagy, acting as gatekeepers of metabolic activity (Warr and Passegué, 2013). Other regulatory pathways are also involved in this process, such as SIRTs, FoxO3A, AMPK, and p53 signaling pathways (Ito et al., 2006; Jang and Sharkis, 2007; Tothova and Gilliland, 2007; Nakada et al., 2010; Taniguchi Ishikawa et al., 2012; Wanet et al., 2015; Jung et al., 2016; Qian et al., 2016;; Zhang et al., 2018).

### 5.3 Extracellular Vesicles

Extracellular vesicles (EVs) play a significant role within the hematopoietic niche in maintaining homeostasis through autocrine and paracrine signaling (Butler et al., 2018). Identification of disease-specific markers in the circulating exosomes aid in the early detection of certain malignancies (Butler et al., 2018). For instance, in chronic lymphoid leukemia, patient-derived exosomes have elevated CD19, CD31, CD44, CD82, HLA-A to D, levels, while low levels of CD21, CD49c, and CD63. In chronic lymphoid leukemia, upregulated levels of CD19 and CD37 have been observed; in AML patients, EVs rich in myeloblastic markers like CD34, CD33, and CD117 have been seen; while multiple myeloma patients have been screened based on EVs with CD38, CD138, CD44 and CD147 markers (Szczepanski et al., 2011; Harshman et al., 2016; Razmkhah et al., 2017). Similarly, EVs are biomarkers to identify patients with aplastic anemia, myelodysplastic syndrome, and sickle-cell anemia, based on their miRNA signatures derived from plasma EVs (Hosokawa et al., 2017; Butler et al., 2018).

For therapeutic purposes, autologous EVs have been obtained from several cell sources, including MSCs, T, and NK cells. MSCs, for example, display immunomodulatory function *via* miR-155 and miR-146 shuttling that inhibits T, B, and NK cells activity since these miRNAs regulate innate immune response by regulating vesicle trafficking (Di Trapani et al., 2016). Similarly, murine MSCs with CD73^+^ and CD34^+^ EVs have been shown to reverse graft-versus-host disease (GvHD) and hindlimb ischemia by suppressing Th1-mediated inflammation (Amarnath et al., 2015). Other cytokines that reduce the inflammatory response within patients include IL-10, TGF-β, and HLA-G. The chronic inflammation caused in Rab27 knockout mice is suppressed by HSCs injection or G-CSF rich EVs (Alexander et al., 2017). However, approaches for organ-specific selective loading need to be optimized before rolling it out for clinical use. Therefore, a practical therapeutic approach, and deeper understanding of the EVs-cellular interaction are needed.

### 5.4 Limitations

Despite the wide-scale clinical application of HSCs targeting many disorders, certain complications are associated with hematopoietic stem cell transplantation (HSCT). Early-onset side-effects include mucositis, hemorrhagic cystitis, GvHD and graft failure, pulmonary complications, thrombotic microangiopathy, and prolonged pancytopenia (Moore, 2021). On the other hand, late-onset complications arise due to chronic GvHD, ocular, endocrine, pulmonary, musculoskeletal, neurological, and immune effects, congestive heart failure, and risk of infections. Late-stage repercussions usually set in after many months or years of HSCT and even report cases of leukemia and myelodysplastic syndrome relapse.

## 6 Conclusion

Hematopoiesis is a complex developmental process regulated by a closely integrated network of TFs, miRNAs, epigenetic modifications, cytokines, and growth factors. These components maintain a fine balance between the self-renewal and differentiation properties of HSCs to generate a functionally stable hematopoietic pool. The BM niche components modulate the heterochromatic expression of multiple genes to maintain HSCs progression into a specific lineage, preventing leukemogenesis occurrence. Dysregulation in these networks leads to a diverse range of hematological malignancies such as ALL, chronic lymphocytic leukemia (CLL), acute myeloid leukemia (AML), chronic myeloid (CML), myeloma, aplastic and sickle-cell anemia, GvHD and lymphoma. In addition to intrinsic factors, various extrinsic factors such as ionization, obesity, and lack of exercise also impact the hematopoietic niche, causes oxidative stress and ablation of long-term renewability of HSCs. Therefore, a more lucid image of the hematopoietic development system is required to strategize natural therapies. Advanced technologies such as single-cell RNA sequencing, multiphoton intravital microscopy, the building of more pragmatic animal models like zebrafish, and high-throughput genetic screening, have deepened our insight into this system. However, a better comprehension of inflammation-upregulated HSCs and EVs trafficking might offer opportunities to discerning novel therapies for HSCs homing in an organ-specific manner to be used in clinical settings. The underlying mechanisms of action of TFs and miRNA of bone marrow niche in interconnecting therapeutic strategies are still poorly understood. It is conceivable that over the years, their role in influencing self-renewal and differentiation will become more apparent as the biological interactions of these microenvironmental factors are unraveled through a continuous moving from bench to bedside and vice versa. Engineered HSCs and controlled *ex vivo* expansion using extracellular vesicles can create HSCs without immunogenicity, making HSCs more suitable for therapeutic purposes as their infusion may be performed across histocompatibility locus antigen barriers without risk of rejection.

## Glossary

AhR: Aryl Hydrocarbon Receptor
AML: Acute Myeloid Leukemia
B-ALL: B-cell Acute Lymphoblastic Leukemia
BM: Bone Marrow
CLL: Chronic Lymphocytic Leukemia
CLPs: Common Lymphoid Progenitors
CMPs: Common Myeloid Progenitors
CSCs: Cancer Stem Cells
EPCR: Endothelial Protein C Receptor
EPO: Erythropoietin
ESCs: Embryonic Stem Cells
EVs: Extracellular Vesicles
FL: Follicular Lymphoma
G-CSF: Granulocyte Colony Stimulating Factor
GMPs: Granulocyte-Macrophage Progenitors
GvHD: Graft-Versus-Host Disease
HPCs: Hematopoietic Progenitor Cells
HSCs: Hematopoietic stem cells
IL: Interleukin
IR: Ionizing Radiation
LT-HSCs: Long-term HSCs
MDS: Myelodysplastic Syndrome
MEPs: Megakaryocyte-Erythrocyte Progenitors
MPN: Myeloproliferative Neoplasm
MPPs: Multipotent Progenitors
MSCs: Mesenchymal Stem Cells
NK: Natural Killer
NOX: NAPDH Oxidase
OXPHOS: Oxidative Phosphorylation
PPAR: Proliferator-Activated Receptor
RA: Retinoic Acid
ROS: Reactive Oxygen Species
scATAC-seq: Single-Cell Assay For Transposase-Accessible Chromatin Using Sequencing
SCF: Stem Cell Factor
scRNA-seq: Single-Cell RNA Sequencing
SLAM: Signaling Lymphocyte Activation Molecule
SR1: Stem-Reginin1
ST-HSCs: Short-term HSCs
T-ALL: T-cell Acute Lymphoblastic Leukemia
TFs: Transcription Factors
TPO: Thrombopoietin
UCB-CD34^+^: Umbilical Cord Blood Derived HSCs
VA: Valproic Acid
